# The potassium channel Ether à go-go is a novel prognostic factor with functional relevance in acute myeloid leukemia

**DOI:** 10.1186/1476-4598-9-18

**Published:** 2010-01-27

**Authors:** Jasmin R Agarwal, Frank Griesinger, Walter Stühmer, Luis A Pardo

**Affiliations:** 1 Max-Planck-Institute of Experimental Medicine, Hermann-Rein-Str. 3, 37075 Göttingen, Germany; 2 Department of Hematology and Oncology, University Medicine Göttingen, Robert-Koch Strasse 40, 37075 Göttingen, Germany

## Abstract

**Background:**

The voltage-gated potassium channel hEag1 (K_V_10.1) has been related to cancer biology. The physiological expression of the human channel is restricted to the brain but it is frequently and abundantly expressed in many solid tumors, thereby making it a promising target for a specific diagnosis and therapy. Because chronic lymphatic leukemia has been described not to express hEag1, it has been assumed that the channel is not expressed in hematopoietic neoplasms in general.

**Results:**

Here we show that this assumption is not correct, because the channel is up-regulated in myelodysplastic syndromes, chronic myeloid leukemia and almost half of the tested acute myeloid leukemias in a subtype-dependent fashion. Most interestingly, channel expression strongly correlated with increasing age, higher relapse rates and a significantly shorter overall survival. Multivariate Cox regression analysis revealed hEag1 expression levels in AML as an independent predictive factor for reduced disease-free and overall survival; such an association had not been reported before. As a functional correlate, specific hEag1 blockade inhibited the proliferation and migration of several AML cell lines and primary cultured AML cells *in vitro*.

**Conclusion:**

Our observations implicate hEag1 as novel target for diagnostic, prognostic and/or therapeutic approaches in AML.

## Background

Acute myeloid leukemia (AML), characterized by strong proliferation of undifferentiated hematopoietic progenitor cells, is the most common type of acute leukemia in adults. AML subtypes are very heterogeneous, with different chromosomal aberrations, therapeutic response and outcome. Age and certain chromosomal aberrations are markers for a good or bad prognosis, but 45% of AML have a normal karyotype and an unclear intermediate prognosis with a five-year survival rate of only 40% [1]. Prognosis factors are crucial for therapy decisions like bone marrow transplantations, which can cure the disease but are not devoid of severe side effects. An increasing number of genetic parameters (mainly gene mutations implicated in hematopoietic differentiation or transcription regulation) are being identified as predictive factors. Examples are FLT3-ITD (Internal Tandem Duplication of Receptor Tyrosine Kinase) or partial tandem duplications of the MLL gene (Mixed Lineage Leukemia), which are associated with a poor prognosis [2,3]. Therapies against certain subtypes with distinct biological features offer noticeably improved outcome, as established for AML M3 with 90% survival, highlighting the need for specific therapeutic regimes [4].

Increasing evidence relates ion channels to cancer pathogenesis and prompts their use in diagnosis and therapy [5]. Voltage-gated K^+^-channels show the highest variability among ion channels with over 70 genes. The channels expressed in a given cell are specific not only for the cell type but also for its physiological status. These channels represent a suitable distinctive element for both healthy and tumor cells and have aroused significant interest in cancer research [5-7]. Our group is specifically interested in the human voltage-gated potassium ion channel ether à go-go 1 (hEag1) because of its pathological expression in tumor cells and its potentially oncogenic properties [8-13]. The physiological expression of hEag1 is largely restricted to the brain where its role is still unknown [14], although transient hEag1 expression induces myoblast fusion and exit from the cell cycle during myoblast differentiation [15]. hEag1 expression and functional characteristics are modulated in neuronal cells throughout the cell cycle [16] and by many factors like the cytoskeleton or calmodulin [8,17]. During neuroblastoma differentiation, hEag1 expression is strongly down-regulated [18].

Non-neural cells aberrantly overexpressing hEag1 acquire phenotypical characteristics of malignancy and induce aggressive tumor growth in immunodeficient SCID mice [12]. hEag1 is significantly overexpressed in many tumor cell lines and more than 75% primary solid tumors from different histological origins like breast, colon or cervix carcinomas [10,19] and sarcomas [11]. Importantly, the channel cannot be detected in the originating normal tissues. hEag1 inhibition by the antihistamine astemizole, the tricyclic antidepressant imipramine or hEag1 specific monoclonal antibodies reduces tumor cell proliferation *in vitro *and *in vivo *[9,11,20-23]. In summary, the expression of hEag1 in many solid tumors is clearly established though its functional role in carcinogenesis or tumor maintenance is still under investigation.

In chronic lymphatic leukemias the related channel HERG (human Eag-related gene, K_V_11.1) could be identified, but no hEag1 expression was detected [24]; it was subsequently assumed that hEag1 had no relevant role in leukemias.

Leukemias and lymphomas are an important and frequent group of tumors with very distinct pathophysiological features in contrast to solid tumors. The goal of the current study was to determine if different types of leukemias share the biological feature of solid tumors to express hEag1. Additionally, we wanted to determine if any potential expression might have functional correlates or even prognostic value. Within leukemias we focused on AML and analyzed hEag1 expression by quantitative real-time PCR (qPCR) and correlated it to several patient characteristics. A possible role of hEag1 in leukemia cell proliferation could be shown by growth and migration inhibition of the hEag1-expressing cell lines PLB-985, K562, UT-7 and HEL and primary clinical samples by the potassium channel blockers astemizole, imipramine, the hEag1 specific monoclonal antibody mAb56 and siRNA knockdown [9]. No involvement of hEag1 expression during HL-60 cell differentiation was detected. Cell cycle related changes and apoptosis induction were analyzed to determine possible inhibitor effects useful for any hEag1-based therapy.

## Results

### hEag1 is frequently expressed in primary myeloid leukemias and leukemia cell lines

As the expression of the hEag1 channel in leukemias is unknown our aim was to study the prevalence of hEag1 in different kinds of leukemias.

**Figure 1:**
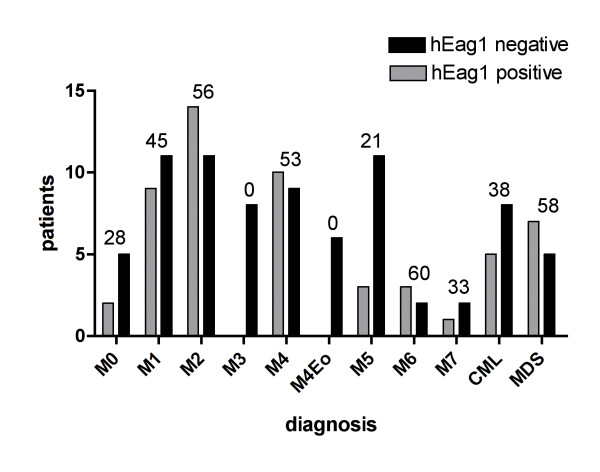
**hEag1 expression patterns in leukemia**. A. Percentage of hEag1 positive patients as a function of cytological diagnosis. The percentage of hEag1 positive samples in each subgroup is given on top of each bar pair. hEag1 expression was subtype dependent in AML.

We determined hEag1 expression in 181 blood or bone marrow samples derived from patients with hematological disorders, including AML, ALL, CML and MDS. Samples with low RNA content (n = 12) or lacking clinical data (n = 15) were excluded from the study. Finally, hEag1 expression was analyzed in 154 samples by qPCR. The results are summarized in Fig. 1 and Table 1. We confirmed that healthy peripheral blood cells do not show detectable hEag1 expression (n = 10).

**Table 1:** Patient characteristics

	Study population	hEag1 positive	hEag1	P-value
			negative	
**No (%)**	118	47 (40)	71	
**Gender, no. of males (%)**	59	23 (50)	36	1.0
**Age, years **median (range)	49(1-89)	60 (1-86)	31 (1-89)	<0.001***
**Age groups, N (%)**				<0.001***
child (1-18 years)	37	5 (13)	32	
adult (19-59 years)	38	18 (47)	20	
elder (60-90 years)	43	23 (53)	20	
**FAB classification, N (%)**				0.32
M0	7	2 (28)	5	
M1	20	9 (45)	11	
M2	25	14 (56)	11	
M3	8	0	8	
M4	19	10 (53)	9	
M4Eo	6	0	6	
M5	14	3 (21)	11	
M6	5	3 (60)	2	
M7	3	1 (33)	2	
unclassified	11	5 (45)	6	
**Cytogenetic analysis, N (%)**				0.11
normal	38	25 (66)	13	
t (8;21)	7	3 (43)	4	
inv (16)	6	0	6	
t (15;17)	8	0	8	
11q23 abnormalities	10	1 (10)	9	
FLT3-ITD	5	4 (80)	1	
chromosome 5 or 7 abnormalities	16	7 (44)	9	
Trisomy 8 alone	6	2 (33)	4	
other single abnormality	6	1 (17)	5	
multiple abnormalities	22	9 (41)	13	
unknown	14	4	10	
**Cytogenetic classification, no. (%)**				0.66
Favorable	16	3 (19)	13	
Intermediate	49	25 (51)	24	
Unfavorable	47	17 (36)	30	
Unknown	7	1	6	
**Stem cell transplantation**	22	5 (23)	17	0.29
No stem cell transplantation	55	19 (34)	36	
unknown	41	22	19	
**CR1 achieved, no. (%)**				0.94
achieved	38	13 (34)	25	
not achieved	27	9 (33)	18	
unknown	53	24	29	
**Relapse, no. (%)**				0.41
early relapse (within 6 months)	10	5 (50)	5	
late relapse	32	11 (34)	21	
no relapse within 3 years	25	3 (12)	22	
unknown	24	13	11	
**Death, no. (%)**				0.64
early death (within 6 months)	26	13 (50)	13	
late death	32	14 (44)	18	
**Disease free survival (patient no.)**	99	37	62	0.0023***
median, months	14	7	22	
disease free at 3 years, no. (%)	28	5 (13)	23 (37)	
**Overall survival (patient no.)**	99	37	62	0.0019***
median, months	24	10	52	
alive at 3 years, no. (%)	35	6 (16)		29 (47)
alive at 5 years, no. (%)	21	2 (5)	19 (31)	
**Therapy protocol**				
AML-BFM-98	33	4	29	
HD 98 A	15	4	11	
AMLCG	12	5	7	
AML-SG 06-04	5	4	1	
AML-SG 07-04	5	3	2	
HOVON-SAKK AML-43	6	4	2	
supportive	9	6	3	
unknown	34	16	18	
**Peripheral blood blasts, % **median (range)	65 (0-100)	42 (0-95)	66 (2-100)	0.39
**Bone marrow blasts, % **median (range)	85 (0-100)	90 (25-98)	85 (0-100)	0.94
**WBC, ×10^9^/l **median (range)	33.7 (0.9-400)	50.8 (2.6-222	32 (0.9-400)	0.76

The table shows the clinical characteristics of all analyzed patients. Percentages refer to the corresponding study population. hEag1 expression is correlated to increased age, unfavorable prognosis, higher relapse rates and strongly shorter overall survival. FAB: French-American-British classification of AML; WBC: white blood cell count; CR1: first complete remission.

Acute lymphoblastic leukemia (ALL) (n = 8) and biphenotypic acute leukemia (n = 3) were hEag1-negative in all samples analyzed.

hEag1 was detected in 5 of 13 cases (38%) of chronic myeloid leukemia (CML), and interestingly, 58% of the samples with myelodysplastic syndrome (MDS; n = 12) expressed hEag1. Many MDS progress into AML, but inducing factors and time until progression are still unknown. One hEag1 positive patient with MDS progressed quickly after two months into an AML M2, another patient died after five months. Two other patients with hEag1 positive MDS were followed for more than one year and two patients for more than two years without any progression. We further followed one patient with AML M4 that was hEag1 positive at first diagnosis. After 14 months, a relapse occurred and the amount of hEag1 expression was strongly increased.

40% of the analyzed AML samples expressed hEag1 (47 of 118 samples). According to the FAB (French-American-British) classification, a subtype-dependent expression pattern was observed: AML FAB M3 (n = 8) and M4Eo (n = 6) were consistently hEag1-negative. Within the other subgroups, hEag1 was expressed in 2 of 7 M0 (28%), 9 of 20 M1 (45%), 3 of 14 M5 (21%), 3 of 5 M6 (60%) and 1 of 3 M7 cases (33%). The most common subtypes M2 and M4 frequently expressed hEag1 (14 of 25 cases (56%) and 10 of 19 cases (53%), respectively) (Fig. 1).

**Figure 2:**
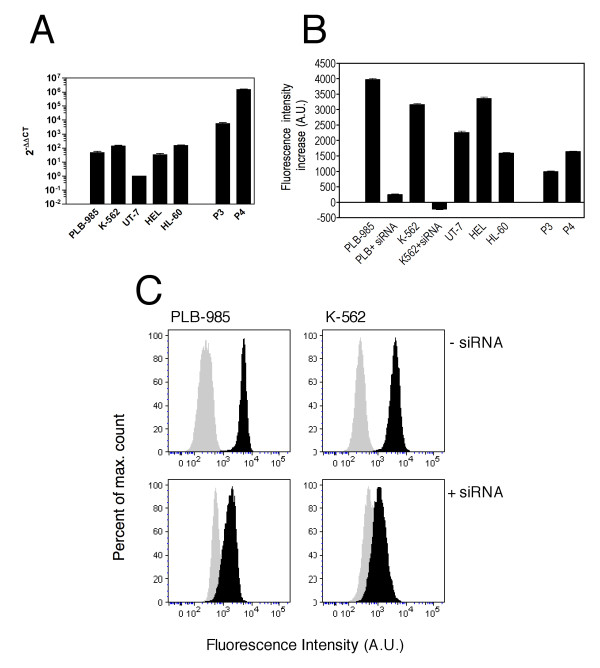
**Expression of hEag1 in leukemia cell lines and primary AML cells**. A: hEag1 mRNA levels determined by real-time PCR, expressed as relative to that in UT-7 cells. B: hEag1 protein content in cell lines and primary cells from hEag1 positive patients measured by flow cytometry. The background rightward shift induced by anti-hEag1 antibody mAb49 in the PCR-negative P2 cells was set as zero value. C: hEag1 siRNA treatment (lower panels) strongly diminished the fluorescence shift attributable to hEag1 expression in PLB-985 and K562 cells. The gray peak indicates control secondary fluorescent antibody staining.

hEag1 expression was also tested by real-time PCR in HL-60, UT-7, K562, PLB-985, HEL, KASUMI and CMK cell lines. hEag1 RNA was detected in K562, PLB-985, UT-7, HL-60 and HEL cells. Primary cells were obtained from the peripheral blood of four AML patients, and hEag1 expression was detected in two of them (P3 and P4, Fig. 2A).

In order to establish specificity of the hEag1 inhibitor effects during proliferation assays, we also determined the expression of HERG (K_V_11.1) in the hEag1 positive cell lines. UT-7 cells expressed equal and K562 cells higher levels of HERG channels compared to hEag1, whereas we did not detect HERG in PLB-985, HL-60 or HEL cells. All four patient samples were negative for HERG expression.

Protein expression was confirmed by flow cytometry using an anti-hEag1 specific monoclonal antibody (mAb49) directed against an extracellular loop of the channel. The results are summarized in Fig. 2B. Some background fluorescence was detected in all cell lines, including hEag1-negative patient samples. For this reason, we subtracted the magnitude of the mean fluorescence intensity (MFI) shift observed in Patient 2 cells (hEag1 negative in PCR) from all measurements. Under these conditions, hEag1 knockdown by siRNA in PLB-985 and K562 cells completely abolished the increase in fluorescence intensity (Fig. 2C), indicating that the MFI shift is due to hEag1 protein expression.

The hEag1 channel is indeed expressed in several cell lines and primary clinical specimens of newly diagnosed myeloid leukemias.

### hEag1 expression in AML is subtype-dependent and correlates with increasing age

Broad statistical analyses were performed for AML because of the relatively high number of samples available. There was no significant correlation between hEag1 expression and initial laboratory parameters such as platelet counts (median, 58 *vs*. 54 × 109/l; p = 0.62), LDH (511 *vs*. 451 U/L; p = 0.24), hemoglobin (9.5 *vs*. 8.8 g/dL; p = 0.36), peripheral blood blasts (42 *vs*. 66%; p = 0.39) and bone marrow blasts (90 *vs*. 85%; p = 0.94). White blood cell counts were higher in hEag1 expressing samples without reaching statistical significance (median, 50.8 *vs*.32 × 10^9^/L; p = 0.76). There was also no difference in gender distribution between hEag1 positive and hEag1 negative patients (p = 1.0).

**Figure 3:**
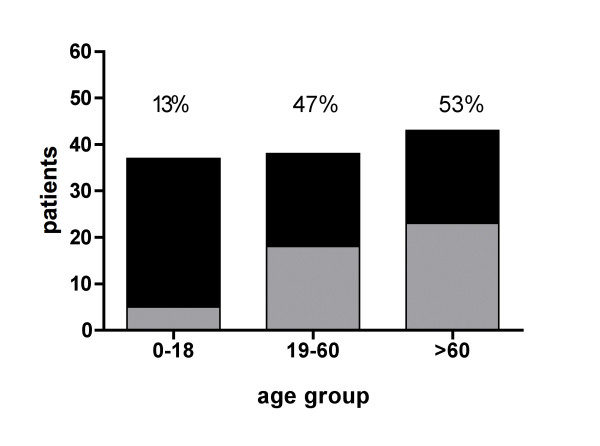
**hEag1 expression correlated to three age groups: 0-18, 19-59 and 60-89 years**. With increasing age hEag1 expression became more abundant.

hEag1 expression was significantly more frequent in older patients (median 60 *vs*. 31 years, p = <0.001). 13% of AML in children (up to 18 years; n = 37) but 47% of adults (19-59 years; n = 38) and 53% of patients above the age of 60 years (n = 43) were hEag1 positive (Fig. 3).

Three prognostic groups can be defined in AML according to cytogenetic aberrations. The favorable group is defined by t(8;21), t(15;17), inv(16) or young age. The intermediate group consists of patients with normal cytogenetics, +8, +21, +22 or -9q, while a complex karyotype, -7, -5, MLL anomalies or FLT3-ITD (31) correlate with poor prognosis. hEag1 expression was often associated with an intermediate prognosis in 25 of 49 patients (51%). In 17 of 47 patients it correlated with an unfavorable prognosis (36%, p = 0.66), while only 3 of 16 patients (19%) with a favorable prognosis profile expressed hEag1. Chromosome 5 or 7 abnormalities (n = 16) or multiple aberrations (n = 22) were often associated with hEag1 expression in 44% and 41%, respectively. Interestingly, 4 of 5 patients (80%) with FLT3-ITD were hEag1 positive.

The highest prevalence of hEag1 was reached in the most frequent subtypes AML M2 and M4 with expression in more than half of the cases. Channel expression significantly correlated with increasing age.

### The hEag1 expression level in primary AML is a novel independent predictive factor for a poor outcome

The frequent expression of hEag1 in AML suggests an involvement of the channel in the pathophysiology of this disease, which might be linked to patient outcome. To test this hypothesis, statistical quantifications were performed.

**Figure 4:**
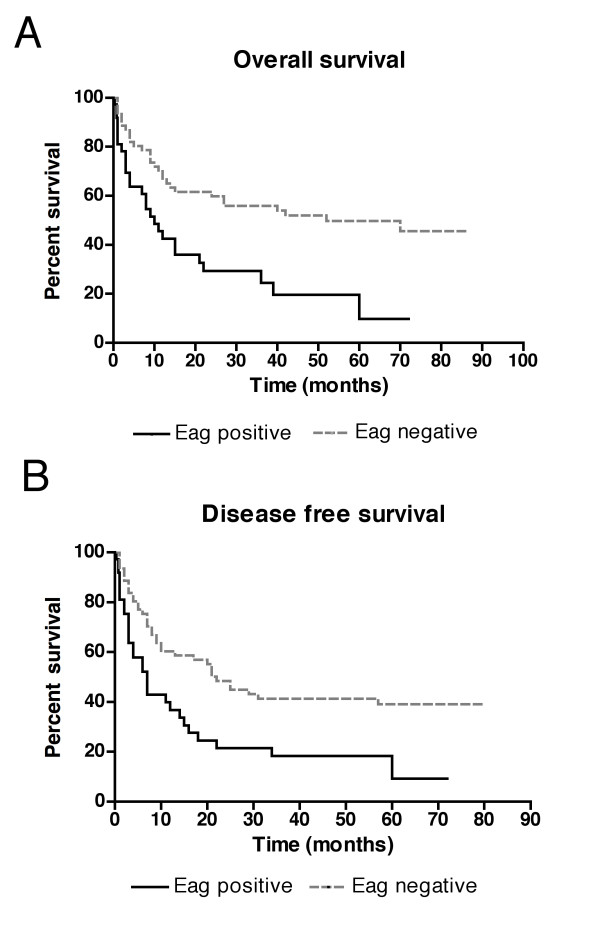
**Shorter survival in hEag1 positive patients**. Kaplan-Meier curves show the disease-free and overall survival of patients expressing (black line) or not expressing (grey dashed line) hEag1. A significantly shorter disease-free (7 *vs*. 22 months, p = 0.0023) and overall survival (10 *vs*. 52 months, p = 0.0019) can be observed in hEag1 positive versus hEag1 negative patients.

Relapse occurred significantly more frequently in hEag1 positive patients both at early (less than 6 months, 26% *vs*. 10%) and late time points (58% *vs*. 44%). The median disease-free survival of hEag1 positive patients was strongly reduced compared to hEag1 negative patients (7 *vs*. 22 months, p = 0.0023). After 3 years, only 17% of hEag1-expressing patients were free of disease, as compared to 41% of hEag1-negative patients. In addition, the overall survival was strongly reduced if leukemic cells expressed hEag1 (median OS, 10 *vs*. 52 months; p = 0.0019). Only 24% of the hEag1-positive patients were alive after 3 years, compared to 54% of the hEag1-negative patients. The estimated five-year survival rate was 15% for hEag1-positive and 48% for hEag1-negative patients (Fig. 4). This shows a clear correlation of hEag1 expression with patient outcome.

To determine if hEag1 expression adds predictive value to age or karyotype alone, we used Cox regression multivariate analysis. hEag1 expression levels, age and karyotype are independent predictive parameters for the overall survival of AML patients (p = 0.044, 0.058 and 0.057, respectively). In a positive/negative thresholded analysis, without taking into account expression level in the positive patients, channel expression did not add predictive value over age alone in the elderly and mid-age patients (older than 65 and 17.28 to 65 years; p = 0.59 and 0.44), but was still highly significant as a bad prognostic factor in young patients (p = 0.0022).

In summary, we conclude that the expression level of hEag1 strongly correlates with shorter survival expectancy in AML and can be used as a novel independent predictive factor, which eventually could be introduced into routine prognosis analysis for advanced grouping and therapy planning of AML patients.

### Functional effects of hEag1 inhibition *in vitro*

**Figure 5:**
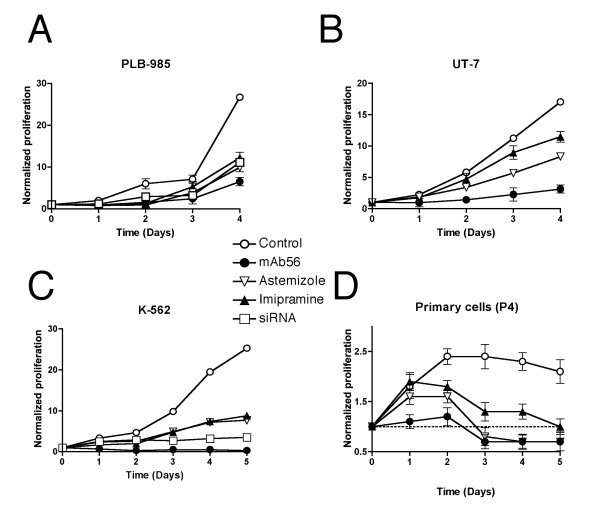
**Effects of hEag1 blockade on proliferation of leukemia cells *in vitro***. Proliferation inhibition of PLB-985 (A), UT-7 (B), K562 (C) and primary cells (P4; D) treated with 20 μM imipramine (filled triangles), 4 μM astemizole (open triangles), 10 μg/mL monoclonal hEag1 antibody mAb56 (filled circles) or 30 nM anti-hEag1 siRNA (open squares) is shown over 4 or 5 days as compared to control cells (open circles). (Mean ± SEM).

Since hEag1 adds independent predictive value in AML, it is conceivable that it might play a functional role in leukemogenesis or disease maintenance. To address this question we studied potential involvements of hEag1 in the growth control, migration properties and differentiation of AML cell lines and primary clinical samples. Proliferation assays were performed with PLB-985, UT-7, K562 and HEL cells. Different concentrations of the hEag1 inhibitors astemizole, imipramine and mAb56 were tested for inhibition of proliferation. None of the inhibitors consistently affected HEL cell proliferation. In contrast, the proliferation of PLB-985, UT-7 and K562 (Fig. 5) cells was strongly inhibited (up to 77% after 4 days, p < 0.05) in a dose-dependent manner by astemizole (0.5-4 μM) or imipramine (5-20 μM). These concentrations correlate well with those described in other cell types [21,23,25]. Among primary clinical samples, only P3 and P4 (hEag1 positive) showed detectable proliferation. Of these, the proliferation of P4 cells was strongly inhibited by all inhibitors (virtually abolished after three days, Fig. 5D)

Previous studies [26] described impairment of leukemia cell proliferation by HERG inhibition. HERG is abundantly expressed in K562 cells and in low amounts in UT-7 cells. It is conceivable that part of the observed effect is due to HERG blockade. This argument is not applicable to PLB-985 or primary cultured cells, where we did not detect any expression of HERG. To clarify the extent of hEag1 implication in the regulation of proliferation of these cell lines, we used the monoclonal antibody mAb56, a very specific inhibitor of hEag1 that does not modify HERG and even not hEag2 activity. 10 or 20 μg/mL mAb56 inhibited PLB-985 by 70%, UT-7 by 80% and K562 proliferation by 98% after 4 days (p < 0.05). The mAb56 abolished the proliferation of P4 primary cells.

Finally, knockdown of hEag1 expression by siRNA in PLB-985 and K562 cells also resulted in up to 80% diminished proliferation over 5 days. (hEag1 knockdown by siRNA was not possible in UT-7 cells.) Representative proliferation curves are shown in Fig. 5. Effective hEag1 knockdown by siRNA was confirmed by flow cytometric detection of cells stained with anti-hEag1 mAb49 (see Fig. 2C). Altogether, our data suggest that hEag1 is implicated in the regulation of leukemia cell proliferation.

**Figure 6:**
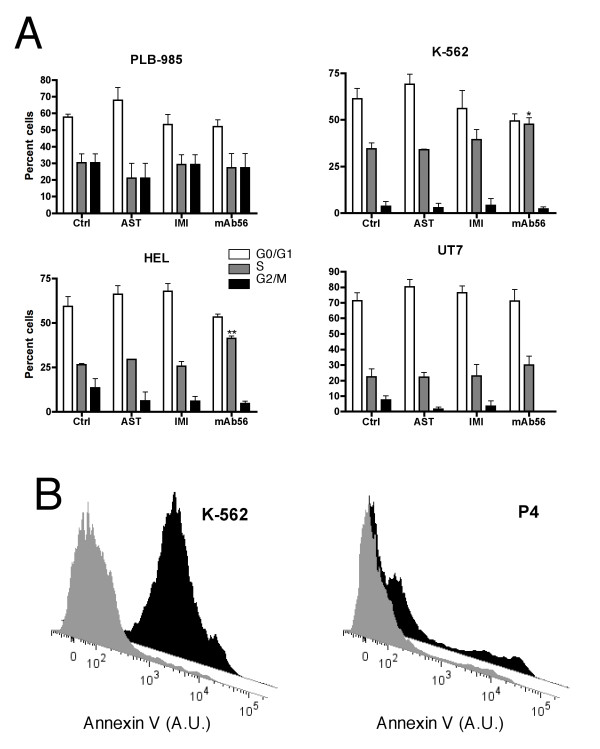
**Cell cycle distribution and apoptosis in the presence of hEag1 inhibitors *in vitro***. A. The indicated cell lines were incubated with 4 μM astemizole (AST), 20 μM imipramine (IMI) or 10 μg/mL anti-hEag1 mAb56 for 2 days. Cell cycle phases were measured after staining with propidium iodide by flow cytometry. Only mAb56 increased significantly the proportion of cells in the S phase in K562 and HEL cells. B. K-562 and primary P4 cells were incubated with 4 μM astemizole or mAb56 antibody for 2 days, respectively and apoptosis was measured by flow cytometry. A clear increase in Annexin V fluorescence (apoptosis) was observed in both determinations.

Proliferation inhibition can be a result of cell cycle arrest. Such an effect has been described for the related ion channel HERG, whose inhibition leads to cell arrest at the G1/S transition [27]. To test a similar scenario for hEag1, we analyzed cell cycle distribution after inhibitor treatments. hEag1 inhibition did not consistently affect the cell cycle distribution of these cell lines (Fig. 6A). Nevertheless, the mAb56 increased the fraction of cells in the S phase in K562 and HEL cells significantly. In primary cultured cells only G0-G1 populations could be detected. hEag1 might be required for the transition between S and M or for DNA synthesis itself as the mAb56 increases the amount of cells in the S phase of the cell cycle in K562 and HEL cells.

Apoptosis induction is a desirable therapeutic effect. For K562 cells, an intense induction of apoptosis was observed after treatment with imipramine and astemizole up to 81% after 1 day (Fig. 6B). Increased apoptosis was not detected in other cell lines, nor was it induced by siRNA or mAb56, indicating that the increased apoptosis is not solely due to hEag1 blockade. In primary AML cells from one patient (P4) apoptosis and necrosis was 2 times increased by 10 μg/mL mAb56 and 20 μM imipramine (Fig. 6B).

**Figure 7:**
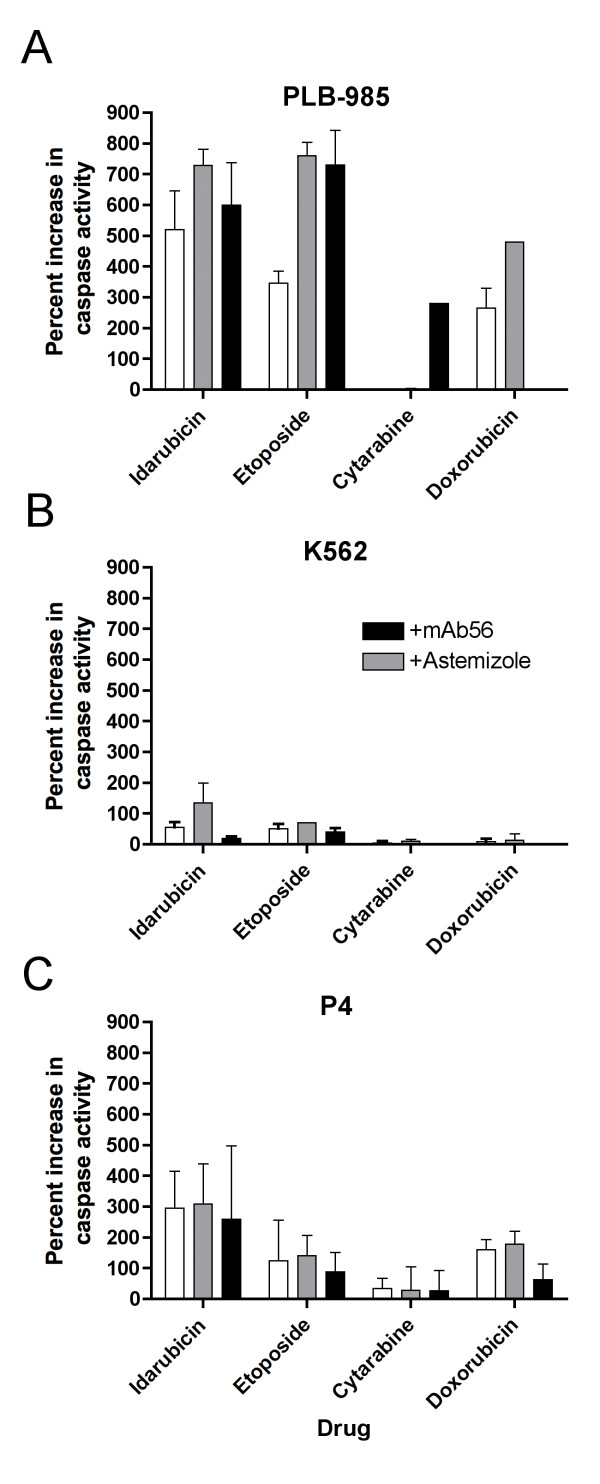
**Induction of apoptosis by chemotherapeutic drugs**. Apoptosis was measured through caspase activation and is represented as relative increase with respect to basal apoptosis in PLB-985 (A), K562 (B) and primary cultured P4 cells (C). Significant increases were observed in PLB-985 cells. In this cell type, Astemizole and mAb56 increased the efficacy of the drugs.

We also tested the effects of hEag1 inhibiting agents in combination with chemotherapeutic agents commonly used for AML induction therapy, cytarabine, etoposide, idarubicin and doxorubicin (Fig. 7). All drugs (except cytarabine) induced some degree of caspase activation in all three cell-types tested, but the effects were intense only in PLB-985 cells (Fig. 7). Apoptosis was strongly increased over basal levels in PLB-985 cells treated with etoposide, doxorubicin and idarubicin. Combination of either of these drugs with astemizole or mAb56 resulted in further increased apoptosis. Cytarabine alone was not able to induce apoptosis in PLB-985 cells, nor did it in combination with astemizole; however, combined with mAb56 antibody it induced caspase activation. K562 cells were relatively resistant to apoptosis induction with any of the drug combinations, although astemizole was still able to increase idarubicin toxicity.

**Figure 8:**
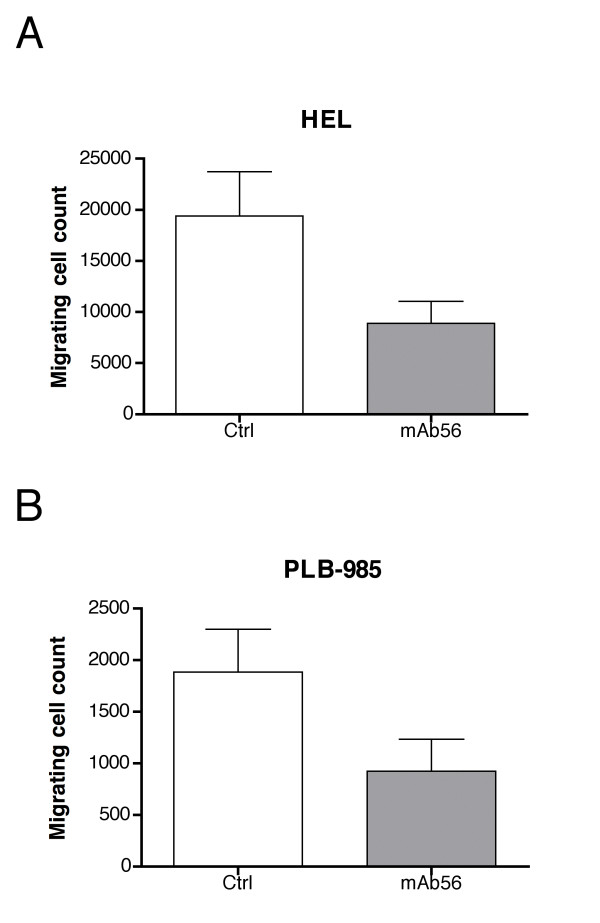
**Inhibition of the migration of cell lines in the presence of hEag1 inhibitors**. HEL and PLB-985 cell migration was reduced by the specific anti-hEag1 mAb56.

Migration is an important feature of malignant cells and its therapeutic inhibition might be critical to avoid metastasis of solid tumors. To fully characterize the capabilities of hEag1 expressing cells, we studied their migration potential and the ability of channel inhibitors to reduce cell migration. K562 and UT-7 cells did not show migration in our experimental paradigm and were therefore not further studied. HL-60 and PLB-958 cells showed limited, although detectable migration, while HEL cells showed much higher migration levels. Inhibition of hEag1 by imipramine and mAb56 (Fig. 8) reduced the migration of HEL and PLB-985 cells up to 65%, indicating an implication of the channel activity in this phenomenon. hEag1-positive primary AML cells showed strong migration without any reduction due to inhibitor treatments.

As transient hEag1 expression is important during differentiation of other cell types like myoblasts, we tested its possible involvement in hematopoietic differentiation. We used the model cell line HL-60, which is arrested at the promyelocytic stage, but can be terminally differentiated over 6 days into granulocytes by retinoic acid. Cell differentiation was shown by Giemsa stain of cell nuclei and an increase of CD38 expression in flow cytometry measurements. We determined hEag1 expression every 2 h during differentiation but did not detect any hEag1 expression at any time during the entire differentiation process (data not shown).

Our functional analyses of AML cell lines and primary AML specimens implicate a role of hEag1 in migration and cell proliferation, without a clear cell cycle arrest. Because hEag1 inhibitors are able to block proliferation and migration they might be useful for therapeutic approaches.

## Discussion

The involvement of hEag1 potassium channels in leukemias had not been systematically studied, although its relevance for many solid tumors is well established. We analyzed the prevalence and prognostic impact of hEag1 expression in 154 patients with newly diagnosed leukemia with special focus on 118 AML patients. In solid tumors, hEag1 is expressed in over 70% of carcinomas and sarcomas [10,11]. Smith *et al*. [24] did not detect hEag1 in CLL, and therefore we did not study CLL samples. In ALL we did not detect any hEag1 expression; although the sample number was low. This might indicate that hEag1 expression is not relevant in lymphatic leukemias.

A completely different scenario applied for myeloid leukemias. Although we detected hEag1 expression in one third of the patients with CML, we did not perform further statistical analyses due to the limited availability of samples. We therefore concentrated on AML, where we found an interesting subtype-dependent hEag1 expression, since one half of the cases of the most common subtypes M2 and M4 expressed hEag1, and this correlated with increasing age, higher relapse rates and a significantly shorter overall survival.

Interestingly, 50% of MDS expressed hEag1. This could represent a similar situation to the report by Farias et al. [19] in cervix carcinoma, where they detected hEag1 expression in cervix carcinomas and cervical hyperplasias that are often pre-forms of carcinomas. If this is also true for AML, hEag1 expression outside the brain might be useful as an early marker for malignant cell transformation, although such a far-reaching conclusion would require the support of prospective studies in a large sample population.

66% of the patients with normal cytogenetic AML expressed hEag1. These patients with a normal karyotype represent a very heterogeneous group with regard to prognosis and therapy response. Several molecular markers like FLT3-ITD, MLL, CEBPA (CCAAT/enhancer binding protein) or BAALC (brain and acute leukemia, cytoplasmic) further characterize some of these patients but there is still highly unmet medical need for other factors to establish a clear prognostic profile in every individual patient [28,29]. It was therefore important to determine if hEag1 expression in AML has prognostic relevance. In other studies, hEag1 expression was associated with an unfavorable outcome in sarcomas, but no multivariate Cox regression analyses were performed, so it is not know if hEag1 expression has any predictive potential in sarcoma [11]. In our study, we observed a clearly decreased survival in hEag1 expressing patients. This could be due to higher expression frequencies in elder patients, since children with AML respond better to therapy than adults. However, multivariate Cox regression analysis determined an independent predictive value for hEag1 expression (p = 0.044), karyotype (p = 0.049), age (p = 0.058) and cytology (p = 0.057), while gender was not predictive (p = 0.20). The median overall survival of 10 months of hEag1 overexpressing patients was strongly reduced compared to hEag1 non-expressing patients with 52 months OS (p = 0.0019). The patient's prognosis is important for therapeutic decisions like bone marrow transplantation, which can prolong the survival or even cure the disease. But it is accompanied with risky side effects that might not be acceptable for patients who anyway have a good prognosis with chemotherapeutic treatment. hEag1 could be used as an additional prognostic factor as it significantly correlates with a bad prognosis. These findings could justify a prospective study to evaluate the use of hEag1 as predictive factor during routine clinical profiling of newly diagnosed AML patients.

Finally, hEag1 expression has been proposed as the basis of a potential new therapeutic target, due to its restricted expression outside the central nervous system and the effects of hEag1 inhibition on the proliferation of several tumor cell types *in vitro *[11,23] and *in vivo *[9,20]. We observed that hEag1 inhibition reduced the proliferation of several AML cell lines. Most available blockers of hEag1 are also effective inhibitors of other channels and enzymes, some of which could be present in the model cells used in this study. However, the observation that the specific anti-hEag1 antibody mAb56 and siRNA show similar strong inhibitory effects as the tested drugs argues for an implication of hEag1 in the proliferation of leukemia cells. The limited number of cell lines expressing hEag1 precluded establishing a reliable correlation between expression levels and impact of channel inhibition on cell proliferation. We could not perform experiments with hEag1 overexpression because we did not achieve reliable transfection efficiencies on leukemia cell lines, either by lipofection or nucleofection, even if control vectors could be successfully transfected, which might indicate deleterious effects of hEag1 overexpression on the cells

One of the primary AML cell preparations with the highest hEag1 content showed comparable behavior to established cell lines. The inhibition achieved through hEag1 inhibitors was even faster and more intense than in cell lines. These results prompt further experiments to establish if leukemias with a particular expression pattern might be suitable targets for anti- Eag1 strategies.

Other members of the Eag K^+^-channel subfamily have been implicated in the pathophysiology of leukemias. HERG channels have been described to be up-regulated in a wide spectrum of hematopoietic malignancies like AML (~70%), CML, ALL or lymphomas, while it is not expressed during physiological hematopoietic development or in normal peripheral blood cells [30]. HERG channels regulate leukemia blast proliferation, improve AML cell migration and invasiveness and correlate with higher relapse and shorter survival [26,31]. The inhibition of HERG reduces leukemia cell growth and arrests cells at the G1/S transition phase [27]. Therefore, HERG channels could have broader therapeutic applications in leukemia than hEag1, but its ubiquitous expression throughout the body and its crucial role in cardiac repolarization are challenging problems for a HERG-based therapy because severe side effects like fatal arrhythmias can occur [32].

In K562 cells, which express high levels of both hEag1 and HERG, we observed a dramatic induction of apoptosis induced by both imipramine and astemizole. It is tempting to speculate that simultaneous inhibition of hEag1 and HERG in those cell types expressing both channels could be an efficacious therapeutic approach.

In combination with routinely used induction therapeutics, the hEag1 blockers astemizole and mAb56 were able to increase the apoptotic response in PLB-985 cells. This could be advantageous by increasing leukemia responsiveness during the critically important induction therapy. However, this effect was cell type specific, indicating that accurate profiling of the AML cells, together with complete toxicity profiles of such drug combinations will be required and have to be critically evaluated in primary cells and animal models.

An increased migration capability is one important sign of malignancy. hEag1 expressing leukemia cell lines show higher migration rates than non-expressing ones. hEag1 inhibitors were able to reduce cell migration. In the context of a therapeutic usage those inhibitors might be important that are able to prevent several features of malignancy like proliferation, migration or functional properties of these transformed cells.

The potential of hEag1 inhibitors for a usage in leukemia treatment has to be carefully further analyzed in primary cells *in vitro *and mouse models *in vivo*.

## Conclusions

We showed that hEag1 is indeed frequently expressed in myeloid leukemia and MDS. In AML it is expressed with high frequency in the most common subtypes and it correlates with increasing age, higher relapse rates and a significantly shorter overall survival. Statistical analyses revealed hEag1 as an independent predictive factor for a worse outcome. Functional studies showed the potential of hEag1 inhibitors to reduce proliferation and migration of AML cell lines and primary cells. Our data suggest a potential for hEag1 as tumor marker for early tumor screening of hematopoietic disorders, as prognostic factor in certain AML types and as therapeutic target of specific inhibitors in the treatment of AML. Antibody-labeled aggressive therapy with radionuclides or toxic molecules could act locally and highly efficiently. More detailed analysis will be performed to establish hEag1 in the diagnosis and treatment of leukemias.

## Methods

### Patients and samples

RNA or frozen cells isolated from the peripheral blood or bone marrow from patients with newly diagnosed AML were obtained from the hematology department of the University Medicine Göttingen. Molecular genetic analyses of patient's blood samples were performed with their informed consent and upon approval by the local ethics committee. Patient details are summarized in Table 1. The median follow up was 4.2 years (range 0.75-7.2 years). Treatment regimes are summarized in Additional File 1.

### Definition of response, relapse and end points

Complete remission (CR) was defined by ≤ 5% bone marrow blasts, neutrophile counts ≥ 1000/μL, platelets ≥ 100,000/μL and lack of extramedullar disease. Cytogenetic remission was defined by normal cytogenetics, and molecular remission by negative molecular studies. Disease-free survival (DFS) was measured from CR to relapse. Overall survival (OS) was defined as time from diagnosis to death irrespective of the cause. Living patients were censored at the date of last follow-up.

### RNA isolation, cDNA synthesis and real time PCR

Mononuclear cells were isolated from peripheral blood or bone marrow by density centrifugation over a Ficoll gradient and RNA was isolated from the buffy coat [33]. Either lysates from frozen cells or total RNA was obtained. Total RNA was isolated from 5 × 10^6 ^cells using the RNeasy mini isolation kit for animal cells (Qiagen, Hilden, Germany) with additional DNAse treatment according to the manufacturer's protocol. cDNA synthesis and real time PCR were performed as previously described [10]. Quantification was performed using the ΔΔC_T _method (cell lines) or after Pfaffl [34].

### Cell culture

The human myeloid leukemia cell lines HL-60 (AML M2, DSMZ ACC 3, Braunschweig, Germany), K562 (CML in blast crisis, ACC 10), PLB-985 (AML M4, ACC 139; this is a subclone of HL-60 cells), HEL (AML M6, ACC 11), CMK (AML M7, ACC 392) and KASUMI-1 (AML M2, ACC 220) were cultured in RPMI1640 medium with 10% fetal calf serum (Invitrogen, Karlsruhe, Germany). UT-7 cells (AML M7, ACC 137) were cultured in alpha-MEM medium with 20% FCS and 5 ng/mL GM-CSF. Cells were maintained at 37°C and 5% CO_2 _in a humidified atmosphere.

Peripheral blood from 4 patients was obtained at the time of initial diagnosis prior to therapy. Primary leukemic blasts were isolated by density centrifugation for 30 min at 800 × *g *(Lymphoprep, Axis-Shield, Oslo, Norway) Cells from the buffy coat were washed three times with PBS and cultured in RPMI1640 medium supplemented with 20% FCS, 1% pyruvate (Sigma-Aldrich, Steinheim, Germany), 1% insulin-transferrin-selenium (ScienCell, Carlsbad, CA) and 1% penicillin/streptomycin (Invitrogen).

Differentiation of HL-60 cells into granulocytes was induced with 2 μM retinoic acid over 6 days [35,36]. During differentiation, samples were obtained every 2 h and analyzed for hEag1 expression up to day 6. Differentiation was monitored by successive Giemsa stains, and confirmed after 6 days by flow cytometry, through up-regulation of CD38 [37].

To measure apoptosis under induction drugs, 2 × 10^5 ^PLB-985, K562 and primary P4 cells in 24-well plates were incubated with 2 μM idarubicin, 20 μM etoposide, 6 μM cytarabine or 4 μM doxorubicin either alone or in combination with 4 μM astemizole or 10 μg/mL mAb56 for 24 h. Apoptosis was assessed with the Caspase-Glo 3/7 Assay system (Promega) measuring luciferase-induced luminescence in a 96-well plate reader according to the manufacturer's instructions.

### Proliferation assays

PLB-985, UT-7, K562, HEL or primary clinical cells were seeded in 96-well plates (2,000 cells/well or 10,000 primary cells/well) and cultured with different concentrations of astemizole (0.5-4 μM), imipramine (5-20 μM) or mAb56 (5-20 μg/mL) for up to 5 days. These inhibitor concentrations are below non-specific cytotoxic doses (5 μM astemizole [21] and 50 μM imipramine [22]). The functional monoclonal anti-hEag1 antibody mAb56 (10 μg/mL) was used as hEag1-specific blocker because it does not affect HERG or hEag2 [9]. Cell proliferation was measured every 24 h by Alamar Blue (Invitrogen) fluorescence after 2 h incubation with the dye in a 1420 Victor^2 ^Multilabel Counter (Ex 544, Em 590 nm; Wallac-Perkin-Elmer, Waltham, MA). The relative proliferation was normalized to control cell growth without inhibitor.

### siRNA

hEag1 silencing was performed using 30 nM siRNA (sense: r(CAG CCA UCU UGG UCC CUU A)dTdT, antisense: r(UAA GGG ACC AAG AUG GCU G)dTdA). Transfection was performed by nucleofection (Amaxa, Lonza, Cologne, Germany) according to the manufactures recommendations with 2 × 10^6 ^K562 or PLB-985. No transfection was achieved in UT-7 cells. Commercial non-targeting scrambled siRNA (Ambion, Darmstadt, Germany) and anti-GAPDH siRNA were used as negative and positive control, respectively. RNA and protein knockdown after 2 days was confirmed by real-time PCR and flow cytometry.

### Migration analysis

3 × 10^5 ^cells were seeded in the upper reservoir of 24-well Boyden chambers in serum-free medium. The lower compartment contained medium with 20% FCS. After 24 h incubation at 37°C, cells in the lower chamber were centrifuged, resuspended in 10 μL medium and counted in a Neubauer chamber.

### Flow cytometry

For hEag1 protein analysis, cells were fixed for 10 min in 10% formalin, quenched with 100 mM glycine, permeabilized for 5 min in 0.1% Triton-X100 and incubated overnight in blocking buffer (0.2% gelatin in PBS). The primary antihEag1 antibody (mAb49) was used at a concentration of 1.3 μg/mL for 1.5 h. Cells were washed three times with blocking buffer and incubated in 1 μg/mL AlexaFluor 488-labeled secondary antibody for 2 h. The whole procedure was performed at 4°C. Finally, cells were washed four times, resuspended in PBS and measured in a BD FACSAria flow cytometer (Becton Dickinson, Heidelberg, Germany). Only single viable cells (as determined by forward-sideward scatter) were gated and further analyzed.

Apoptosis analysis was performed using Annexin V-AlexaFluor 488 or 680 (Vybrant Apoptosis Assay Kit #2, Invitrogen) and 100 μg/mL propidium iodide according to the manufacturer's instructions.

For cell cycle determination, cells were washed once with PBS and incubated for 15 min in 50 μg/mL propidium iodide, 0.3% saponine and 100 U/mL RNAse A in PBS at 4°C. Flow cytometry measurements were performed within 1 hour, measuring less than 200 cells/second. Data were analyzed with FlowJo software (TreeStar, Ashland, OR).

Differentiated HL-60 cells were stained with 25 μg/mL anti-CD38 antibody per 10^6 ^cells in 100 μL final volume. After 20 min incubation at room temperature, cells were washed and resuspended in PBS.

### Statistical analysis

Estimated probabilities of OS and DFS and time-to-event curves were constructed according to the Kaplan-Meier method and were compared with the log-rank χ^2 ^test. Group data are expressed as mean ± SD. Student's t-test was used for single comparison at 95% confidence. Prognostic factors were examined by multivariate Cox proportional Hazards Survival Regression analysis [38]. Patients with CML or MDS were not statistically analyzed because of small sample numbers.

## Competing interests

WS and LAP are shareholders at iOnGen AG.

## Authors' contributions

JRA, WS and LAP designed research, FG contributed patient samples, JRA performed the experiments, JRA and LP analyzed the data and all authors cooperated in writing the manuscript. All authors read and approved the final manuscript

## Supplementary Material

Additional file 1 **Therapy protocols**. The table summarizes the therapeutic regimes of patients studied. Click here for file
