# Supplementary material for: The potassium channel Ether à go-go is a novel prognostic factor with functional relevance in acute myeloid leukemia
Source: Mol Cancer. 2010 Jan 27;9:18. doi: 10.1186/1476-4598-9-18 (PMC2835655; doi:10.1186/1476-4598-9-18)
Supplement: Additional file 1 — Therapy protocols. The table summarizes the therapeutic regimes of patients studied. [file 1476-4598-9-18-S1.DOC]

**Additional files**

**Additional file 1 – Therapy protocols**

The table summarizes the therapeutic regimes of patients studied.

ICE: idarubicine, cytarabine, etoposide; TAD: thioguanine, cytarabine, daunorubicine; A: cytarabine; ATRA: all-trans retinoic acid; By: busulfane; C: cyclophosphamide; D: daunorubicine; E: etoposide; Gem: gemtuzumab I: idarubicine; M: mitoxantrone; MTX: methotrexate; S: amsacrine; P: Pegfilgrastim; T: thioguanine; H: high dose; auto-SCT: autologous stem cell transplantation; allo-SCT: allogenic stem cell transplantation; HR: high risk; ^1^ all patients with AML FAB M3 were treated with ATRA; ^2^ after induction I; ^3^ cranial irradiation after consolidation; ^4^ if CNS leukemia: Cyt-A, MTX intrathecal; ^5^ randomized as standard consolidation or block therapy; ^6^ if HLA-identical sibling, HR or relapse; ^7^ HD98B: patients > 60 years, lower toxicity regime, ± ATRA; ^8^ if HR or after relapse; ^9^ if no donor: HAM, auto-SCT; ^10^ patients > 60 years: no SCT, no second HAM if CR after induction I; ^11^ patients > 60 years, lower toxicity regime; ^12^ randomization to different time regiments; ^13^ allo-SCT in case of HLA-identical sibling

| **Therapy ^1^** | **AML-BFM-98 ^3^** | **HD 98 A ^7^** | **AMLCG ^10^** | **AML-SG**  **06-04 ^11^** | **AML-SG**  **07-04 ^12^** | **HOVON-SAKK**  **AML-43 ^11^** |
| --- | --- | --- | --- | --- | --- | --- |
| Induction I,II | AIE, HAM  ± G-CSF | A: ICE, ICE  B: ICE,  A-HAM | A: TAD, HAM  ± G-CSF  B:HAM, HAM  ± G-CSF | ICE, ICE | ICE,  ICE ± ATRA | AD, AS  ± G-CSF |
| Consolidation | AI, HAM ^4,5^, HAE  ± cranial irradiation | A: HAM  B: auto-SCT | A: TAD  B: By, C,  auto-SCT | A-HAM, AIE | 3x HA, P,  G-CSF  ± ATRA | A: ME  B: By, C  ± auto-SCT^13^ |
| Maintenance | AT for 1 year | no | A: AD-AT-  AC-AT  for 3 years | no | no | A: no  B: Gem |
| SCT ^2^ | By, C, allo-SCT^6^ | yes ^8,9^ | optional ^8^ | optional ^8^ | yes | B: yes |
